# Supplementary material for: Large Seebeck effect by charge-mobility engineering
Source: Nat Commun. 2015 Jun 25;6:7475. doi: 10.1038/ncomms8475 (PMC4491185; doi:10.1038/ncomms8475)
Supplement: Supplementary Information — Supplementary Figures 1-5, Supplementary Notes 1-4 and Supplementary References [file ncomms8475-s1.pdf]

Supplementary Figures

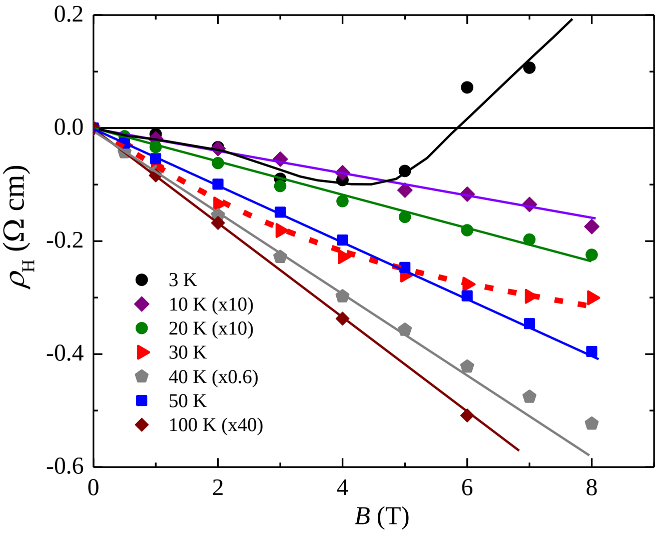

Supplementary Figure 1. Isothermal Hall resistivity ρ_H as a function of magnetic field B at selected temperatures. The dashed line is a two-band fit to $\rho_H(B)$ at T = 30 K (see Supplementary Note 1). Solid lines are a guide for the eyes.

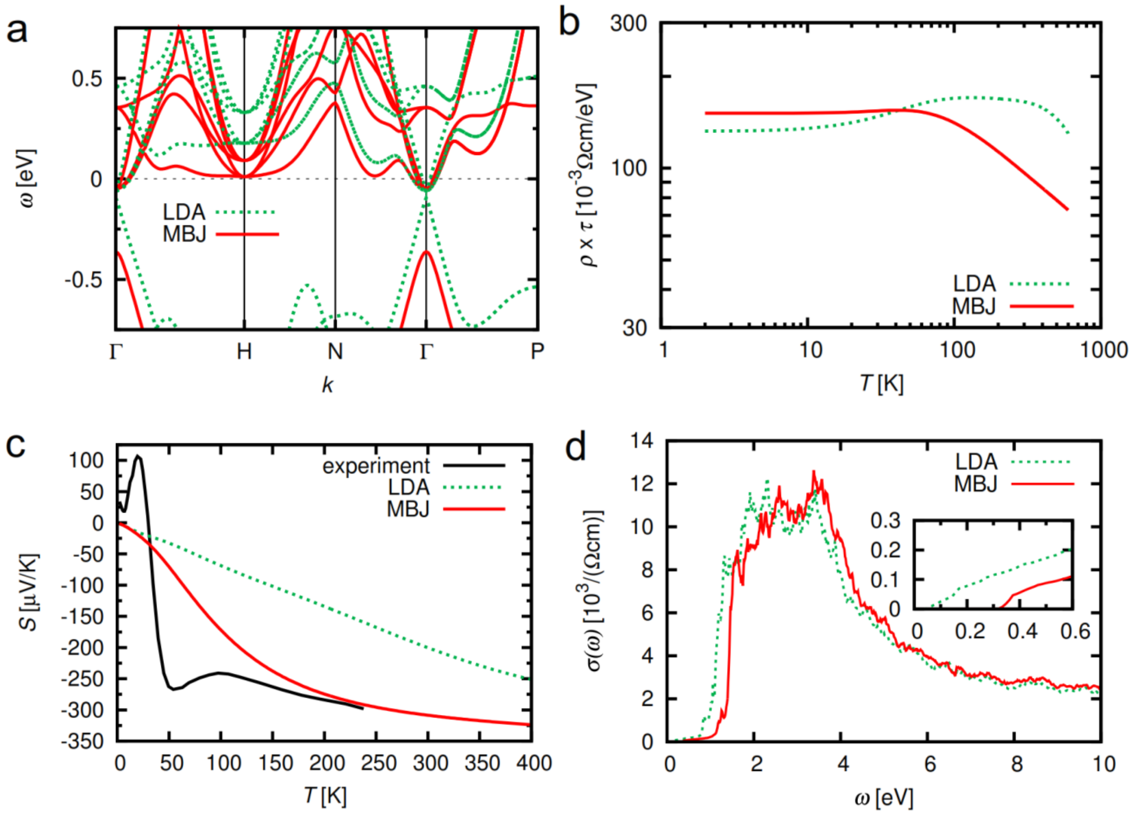

Supplementary Figure 2. Electronic structure and calculated transport properties for $Co_{0.999}Ni_{0.001}Sb_3$. (**a**) Band-structure within LDA and MBJ, with the doping being simulated within VCA. (**b**) Resistivity scaled with the scattering time τ. (**c**) Calculated Seebeck coefficient in comparison with our experimental result. (**d**) Optical conductivity of stoichiometric $CoSb_3$. See Supplementary Note 3 for details.

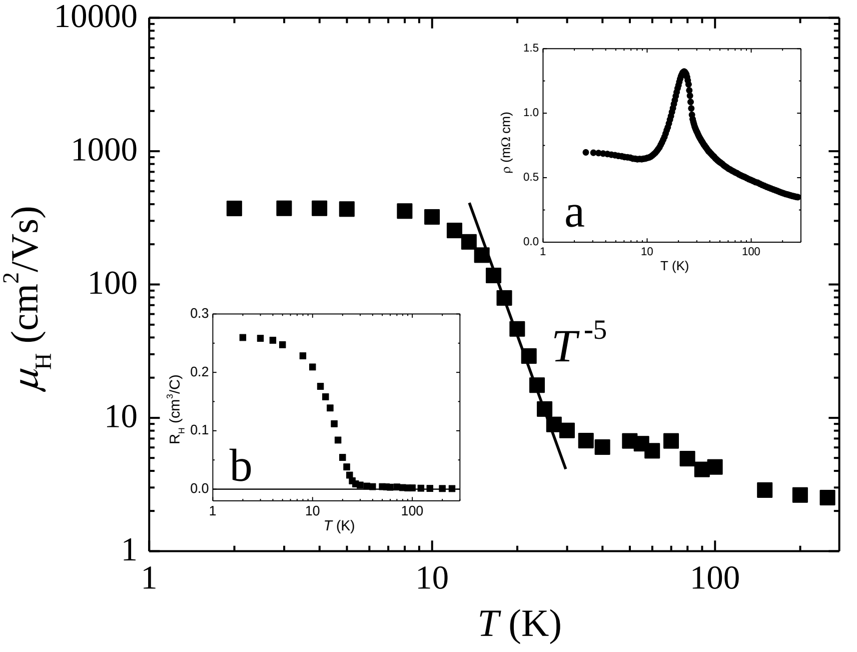

Supplementary Figure 3. Electrical transport coefficients of $CeRu_2Al_{10}$. Main panel: Hall mobility μ_H as a function of temperature. Inset (**a**): electrical resistivity $\rho(T)$. Inset (**b**): Hall coefficient $R_H(T)$.

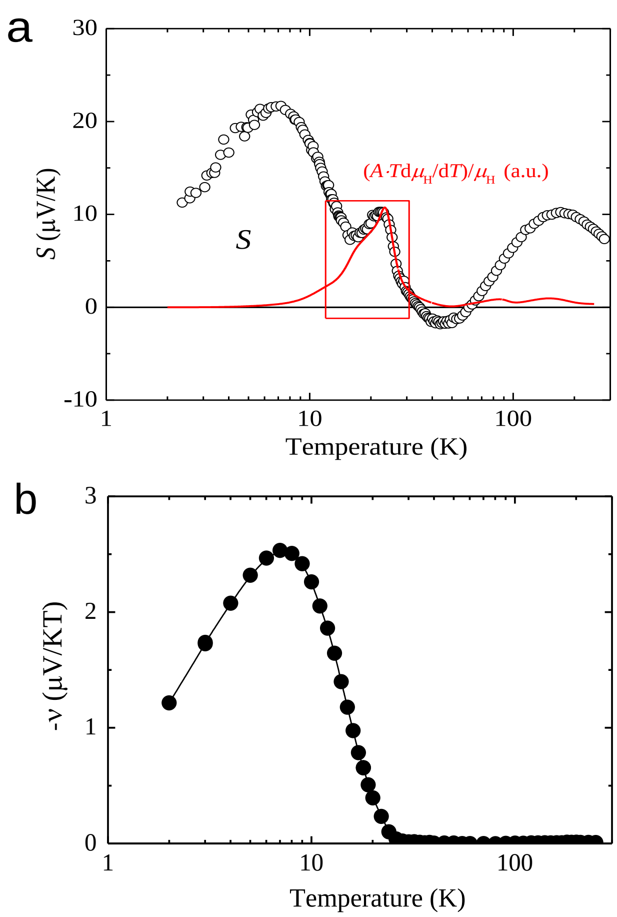

Supplementary Figure 4. Seebeck and Nernst coefficients of $CeRu_2Al_{10}$. (**a**) Seebeck coefficient S and the estimated Seebeck coefficient driven by the mobility mismatch, S_τ = $(AT\mathrm{d}\mu_H/\mathrm{d}T)/\mu_H$, shown in arbitrary units (red line). See main text for details of this estimation. Note that the latter ratio can accurately reproduce the measured Seebeck anomaly emerging in the vicinity of the mobility mismatch. (**b**) Nernst coefficient ν as a function of temperature.

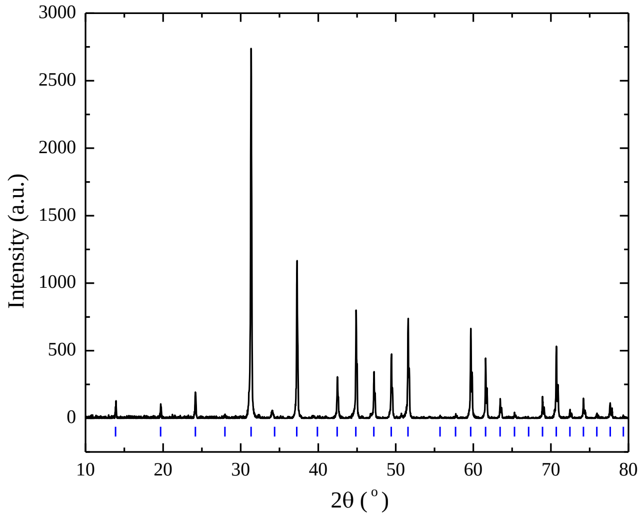

Supplementary Figure 5. Powder X-ray diffraction for $Co_{0.999}Ni_{0.001}Sb_3$. All the peaks can be indexed by the body-centered cubic skutterudite structure with space group *Im-3* (vertical bars at the bottom).

Supplementary Note 1. Two band nature of $Co_{0.999}Ni_{0.001}Sb_3$

The evolution of the multiband nature in the current sample of $Co_{0.999}Ni_{0.001}Sb_3$ can be inferred from the isothermal Hall resistivity $\rho_H(B)$ as a function of temperature (cf. Supplementary Figure 1). Above 50 K, ρ_H is a linear function of the magnetic field B, suggesting a one-band description to be adequate. With lowering temperature, $\rho_H(B)$ starts to deviate from linearity at about 40 K, i.e., the position of both the $R_H(T)$ maximum and the $\rho(T)$ hump, as seen in the Figures 3a and 3b in the main text. The nonlinearity of $\rho_H(B)$ is most obvious at approximately 30 K. By fitting the $\rho_H(B)$ curve at T = 30 K to a two-band model [1], we obtained the Hall coefficient and mobility of the two sub-bands to be $R_1 = -2.34\times10^2$ cm^3/C, $R_2 = -1.54\times10^6$ cm^3/C, μ_1 = 15.3 cm^2/Vs, μ_2 =1840 cm^2/Vs, respectively (cf. the dashed line in Supplementary Figure 1). Here the low-mobility sub-band starts to dominate the electrical transport due to the much larger number of relevant carriers ($n_1 = 1/e|R_1|$). In support of this argument, the Hall coefficient and Hall mobility estimated for T = 30 K within the one-band model are very close to those of the low-mobility sub-band, as seen in Figure 3a of the main text. Decreasing the temperature to below 25 K, a B-linear $\rho_H(B)$ curve is restored (see, for example, the linear $\rho_H(B)$ variation for T = 20 K) and persists until slightly below 10 K. Further lowering temperature results in a more complex $\rho_H(B)$ profile, hinting at more than two bands being involved in the transport, which is beyond our focus of this work.

The hatching between 25 K and 40 K in Figures 2 and 3a in the main text denotes the temperature range where two-band effects have to be considered. Nevertheless, it should be stressed that this effect is actually not essential to our discussion on the origin of the positive $S(T)$ peak emerging below 50 K: A two-band analysis between 40 K and 25 K indicates both bands being of *electron-like* character, with one band apparently being dominating, as discussed above. The argument of two electron-like bands in the temperature interval between 25 and 40 K is in agreement with the analysis of Dyck et al. [2] based on the temperature-dependent ρ and R_H behaviors. In ref. 2, the authors obtained a compensation ratio $K = N_a/N_d$ to be less than 0.5, which indicates the net impurity carriers to be of n type (donor). The fact that both bands are of electron-like character also lends support to our

exclusion of phonon-drag effect from the possible origins of the positive Seebeck anomaly at low temperatures. In view of the sublinear $\rho_H(B)$ curves above 10 K and for clarity of our discussion, in this manuscript, we proceed with the analysis by employing a one-band approximation. This is validated for at least the low magnetic field region ($B \leq 2$ T), where the Nernst effect was measured and the Hall coefficient was estimated.

Supplementary Note 2. Correlation between S_τ, ν and μ_H

Assuming that the one-band approximation applies, the tangent of the Hall angle $\tan\theta_H$ is proportional to τ or μ_H: $\tan\theta_H = eB\tau/m^* = \mu_H B$. As discussed in ref. 3, considering Equation 1 in the main text and taking into account the above relation, one may decompose the Mott expression (Equation 2 in the main text) to

$$
\begin{aligned}
S &= \pm\frac{\nu}{\mu_H} + \left(-\frac{\pi^2}{3}\frac{k_B^2 T}{e}\frac{\partial \ln N}{\partial \varepsilon}\Big|_{\varepsilon=\varepsilon_F}\right) \\
&= S_\tau + S_N,
\end{aligned} \tag{1}
$$

by which, the longitudinal and transverse thermoeletric transport coefficients (i.e., S and ν) are explicitly linked to each other. Apparently, in the current discussion, the charge Hall mobility μ_H as used in this context is preferable to the charge drift mobility, as μ_H (=$\tan\theta_H$/B) directly enters the equation for the Nernst coefficient (cf. Equation 1 in the main text). The first term $\pm\ \nu/\mu_H$ represents the Seebeck effect derived from the asymmetric relaxations of relevant charge carriers, i.e., S_τ. Here the signs, + and −, differentiate the case for hole- and electron-like charge carriers. Note also that the Nernst coefficient ν already includes a sign, which depends on the sign of $d\mu_H/dT$ rather than that of the charge carrier itself (cf. Table 1 in the main text). Therefore, it is obvious that the finally generated S_τ takes a sign according to that of both $d\mu_H/dT$ and the relevant charge carriers. Interestingly, ν and S_τ are simply linked to each other by μ_H, as is the case for ρ_H and ρ. An enhanced ν combining a small μ_H is preferable for a significant S_τ.

Supplementary Note 3. Electronic structure and transport calculations

In order to get theoretical insight into effects of the density of states of Ni-doped $CoSb_3$, we have performed state-of-the-art electronic structure calculations within density functional theory (DFT), as implemented in the wien2k package [4]. Previous electronic structure calculations characterized $CoSb_3$ as a narrow gap semiconductor, with the value of the charge gap varying from 0.05 to 0.22 eV. This large variance originates from the use of different exchange-correlation potentials in the DFT, the slightly different position of the Sb atoms within the unit-cell and, in particular, whether using the experimental or DFT-relaxed lattice constant [5-8].

Here, we use the experimental crystal structure of Ref. 9 with lattice constant a = 9.0347 Å, and apply DFT in the local density approximation (LDA). However, as is common knowledge, the LDA does not sufficiently include exchange effects, resulting in a consistent underestimation of gaps in semiconductors. To address this issue, we also employ the modified Becke-Johnson (mBJ) potential [10] that was found to often match the accuracy of more costly many-body perturbation theories for semiconducting materials. We neglect in all cases the influence of spin-orbit coupling effects.

For stoichiometric $CoSb_3$ (not shown), our LDA calculations yield a charge gap of about 0.04 eV, reproducing previous results [6]. The use of the mBJ functional results in a notable magnification of the gap: We find a value of 310 meV, in good agreement with experimental activation energies [11,12]. We simulate the Ni-doping within the virtual crystal approximation (VCA). For the $Co_{0.999}Ni_{0.001}Sb_3$ composition that we measured experimentally, this yields the band-structures shown in Supplementary Figure 2a. These bands are very close to those without doping, albeit rigidly shifted downwards. Crucially, we observe that the mBJ electronic structure is far more particle-hole asymmetric than the LDA one, owing to a notable rearrangement of conduction bands, as well as the increased gap below the conduction states. We note that there will be a competition [13] between exchange effects (treated effectively within the mBJ) that widen the gap, and dynamical correlations (neglected in DFT) that shrink the gap. Given the apparent influence of impurity bands in the experimental resistivity, an ultimate assessment of the size of the gap will require spectroscopic techniques that e.g. access the optical conductivity. For future reference, we report in Supplementary Figure 2d the theoretical optical conductivity of stoichiometric $CoSb_3$.

Using the implementation described in ref. [14], we finally compute transport properties

within Kubo linear response theory. Supplementary Figure 2b displays the results for the resistivity scaled with the constant scattering time τ. Note that in the one-particle picture, the conductivity basically scales with the scattering time τ, while the Seebeck coefficient is independent of τ. Numerically, we employ a constant inverse scattering time of 8 meV. With the chemical potential residing inside the conduction bands for the Ni-doped case, the system is metallic. However, given the shallow density of states around the Fermi level, the high temperature conduction is dominated by activated transitions across the pseudo gap, yielding an overall insulating-like drop in the resistivity above 70 K within mBJ. Below 70K, the theoretical resistivity multiplied with the scattering time becomes flat in temperature. We take this as an indication that the temperature profile of the mobility, which is discussed as the driving force of the Seebeck anomaly at low temperatures, is also at the origin of the low-temperature characteristics of the experimental resistivity.

Supplementary Figure 2c displays the theoretical Seebeck coefficient in comparison to the experimental results of the main manuscript. As expected from the band structure, the theoretical Seebeck coefficient is negative for all temperatures, and significantly larger in magnitude within mBJ than in LDA. Notably, the mBJ thermopower approaches the experimental result with increasing temperature. Quantitative agreement is reached above 100 K, i.e., at temperatures far from the regime in which we evidenced the mobility anomaly. This quantitative congruence in the high temperature regime suggests that the mBJ approach correctly captures the essence of the intrinsic electronic structure of $CoSb_3$ that is responsible for the one-particle contribution S_N to the Seebeck effect introduced in the main text. The qualitative discrepancy at temperatures below 100 K, however, points to sizeable contributions to the Seebeck coefficients from effects not contained in the (intrinsic) band-structure. This observation further strengthens the proposal that the anomalous Seebeck effect at low temperatures in $Co_{0.999}Ni_{0.001}Sb_3$ is driven by changes in charge relaxation processes, describable by a gradient in the carrier mobility. We note that the shallow feature of the measured $S(T)$ curve in the temperature window 50 – 100 K, which is not attended to in the main text, can actually also find a weak counterpart in the calculated ratio $-\nu/\mu_H$ (cf. Figure 2 in the main text), hinting at a probable involvement of the charge-mobility driven S_τ. Nevertheless, we would leave this feature open, as a deeper in-gap level can also produce such a feature in a semiconductor.

Supplementary Note 4. Unusual Seebeck effect in $CeRu_2Al_{10}$

$CeRu_2Al_{10}$ is a Kondo lattice compound characterized by hybridization of conduction bands with the localized *f* state of Ce ions [15]. While most such compounds have a metallic ground state with a largely renormalized effective electron mass (namely, heavy fermion), some of them are known as heavy-fermion semiconductors or Kondo insulators [16]. $CeRu_2Al_{10}$ is intriguing in that this system experiences a “hidden” order phase transition at around T_0 = 27 K, which separates two distinct regions (cf. Supplementary Figure 3). At higher temperatures, the system behaves like a heavy-fermion semiconductor, whereas at lower temperatures, it is metallic with largely enhanced charge mobility. The nature of the phase transition is still in debate. While neutron powder diffraction and muon spin relaxation have revealed a long-ranged antiferromagnetic ordering of Ce sublattice with a strongly reduced magnetic moment of 0.34 μ_B [17], NMR and NQR experiments failed to capture such an ordering [18]. A sensitive instability of the ordered moment wave vector [19] and the phase transition in $CeRu_2Al_{10}$ being poised at a local-itinerant electron crossover [20] are thought to be the driving physics and are currently being investigated.

The thermoelectric transport of this compound is intriguing as well, with multiple extrema in the $S(T)$ curve, cf. Supplementary Figure 4a. Nevertheless, such behavior is still typical of heavy fermion compounds. For instance, it is very similar to that of the heavy-fermion $CeRu_2Si_2$ [21], except for the abrupt peak at slightly below the abnormal phase transition T_0, namely $T \approx 22$ K, as highlighted in the red frame in Supplementary Figure 4a. In this work, we found that this is exactly the temperature window where a large charge-mobility mismatch occurs (cf. main panel of Supplementary Figure 3). Below we demonstrate that this extraordinary peak in the $S(T)$ curve is derived from the large charge-mobility gradient, in analogy to the discussion made in the main text for Ni doped $CoSb_3$.

The sample employed in this work is a polycrystalline sample prepared by arc-melting the stoichiometric starting materials following ref. 15. As shown in the inset (a) of Supplementary Figure 3, $\rho(T)$ reveals a prominent peak at about 22 K, coinciding with the position of the extraordinary Seebeck peak. Note, however, that there is no phase transition occurring at 22 K since the “hidden” order phase transition is already fully accomplished at T_0. The Hall coefficient $R_H(T)$ is positive in the whole temperature range investigated and increases abruptly below T_0 (cf. inset (b) of Supplementary Figure 3). No obvious nonlinearity in the Hall resistivity vs magnetic field was observed over the whole temperature range investigated, justifying the application of a one-band approximation [15]. Similar to the

Ni-doped $CoSb_3$ introduced in the main text, a dramatic mobility change as a function of temperature ($\mu_H \sim T^{-5}$) is observed from T_0 down to about 10 K, as shown in the main panel of Supplementary Figure 3. Interestingly, the anomalous peak in the Seebeck coefficient occurs right in the middle of this temperature range.

In agreement to the enhanced Hall mobility and the Kondo scattering effect in $CeRu_2Al_{10}$, a large enhancement of the absolute values of the Nernst coefficient $\nu(T)$ is observed below T_0 (cf. Supplementary Figure 4b). It is worth to note that, here the $\nu(T)$ values are negative. By contrast, considering the abrupt negative mobility gradient in $CeRu_2Al_{10}$ and the sign rules given in Table 1 in the main text, a positive $\nu(T)$ peak is expected at around 20 K in response to the mobility mismatch. Nevertheless, we found that the expected Seebeck coefficient from the mobility mismatch, as estimated following $S_\tau = \nu/\mu_H = (AT\mathrm{d}\mu_H/\mathrm{d}T)/\mu_H$, clones the measured Seebeck anomaly (the red frame in Supplementary Figure 4a). As discussed in the main text, here the numerator $AT\mathrm{d}\mu_H/\mathrm{d}T$ denotes the anticipated Nernst response from the mobility mismatch, which, in the case of $CeRu_2Al_{10}$, is most probably masked by the enhanced, negative Nernst background in the high-mobilty Kondo regime below T_0. On the other hand, the Seebeck coefficients outside the red frame (Supplementary Figure 4**a**) can actually be qualitatively accounted for by the Kondo scattering scenario (not shown here). This has already been the focus of ref. 3.

Supplementary References

1. Arushanov, E., Kloc, Ch., Hohl, H. and Bucher, E. The Hall effect in β-$FeSi_2$ single crystals. J. Appl. Phys. **75**, 5016-5019 (1994).
2. Dyck, J. S., Chen, W., Yang, J., Meisner, G. P. and Uher, C. Effect of Ni on the transport and magnetic properties of $Co_{1-x}Ni_xSb_3$. Phys. Rev. B **65**, 115204 (2002).
3. Sun, P. and Steglich, F. Nernst effect: Evidence of local Kondo scattering in heavy fermions. Phys. Rev. Lett. **110,** 216408 (2013).
4. Blaha, P., Schwarz, K., Madsen, G.K.H., Kvasnicka, D., Luitz, J. Wien2k, An augmented plane wave plus local orbitals program for calculating crystal properties (Vienna University of Technology, Austria, 2001).
5. Sofo, J. O. and Mahan, G. D. Electronic structure of CoSb3: A narrow-band-gap semiconductor. Phys. Rev. B **58**, 15620-15623 (1998).
6. Singh, D. J. and Pickett, W. E. Skutterudie antimonides: Quasilinear bands and unusual transport. Phys. Rev. B **50**, 11235-11238 (1994).
7. Koga, K. Akai, K., Oshiro, K. and Matsuura, M. Electronic structure and optical

properties of binary skutterudite antimonides. Phys. Rev. B **71**, 155119 (2005).
8. Wei, W., Wang, Z. Y., Wang, L. L., Liu, H. J., Xiong, R., Shi, J., Li, H. and Tang. X. F. Electronic structure and transport coefficients of binary skutterudite antimonide. J. Phys. D: Appl. Phys. **42,** 115403 (2009).
9. Kjekshus, A. and Rakke, T. Compound with the skutterudie type crystal structure. III. Structural Data for arsenides and antimonides. Acta Chem. Scand. A **28**, 99-103 (1978).
10. Tran, F., Blaha, P., Schwarz, K., Novák, P. Hybrid exchange-correlation energy functionals for strongly correlated electrons: Applications to transition-metal monoxides. Physical Review B **74**, 155108 (2006).
11. Wojciechowskia, K. T., Tobola, J., Leszczynskia, J. Thermoelectric properties and electronic structure of $CoSb_3$ doped with Se and Te. J. Alloys Comp. **361**, 19–27 (2003).
12. Mandrus, D., Migliori, A., Darling, T.W., Hundley, M.F., Peterson, E.J., and Thompson, J.D. Electronic transport in lightly doped $CoSb_3$. Phys. Rev. **B** 52, 4926-4931 (1995).
13. Tomczak, J. M., Casula, M., Miyake, T., and Biermann, S. Asymmetry in band widening and quasiparticle lifetimes in $SrVO_3$: Competition between screened exchange and local correlations from combined GW and dynamical mean-field theory GW+DMFT. Phys. Rev. B **90**, 165138 (2014).
14. Tomczak, J.T., Haule, K., Miyake, T., Georges, A. and Kotliar, G. Thermopower of correlated semiconductors: Application to $FeAs_2$ and $FeSb_2$. Phys. Rev. B **82**, 085104 (2010).
15. Strydom, A. M. Thermal and electronic transport in $CeRu_2Al_{10}$: Evidence for a metal-insulator transition. Phys. B **404**, 2981-2984 (2009).
16. Riseborough, P. S. Heavy fermion semiconductors. Adv. Phys. **49**, 257-320 (2000).
17. Khalyavin, D. D. et al. Long-range magnetic order in $CeRu_2Al_{10}$ studied via muon spin relaxation and neutron diffraction. Phys. Rev. B **82**, 100405(R) (2010).
18. Lue, C. S. et al. Transport, thermal, and NMR characteristics of $CeRu_2Al_{10}$. Phys. Rev. B **82**, 045111 (2010).
19. Bhattacharyya, A. et al. Anomalous change of the magnetic moment direction by hole doping in $CeRu_2Al_{10}$. Phys Rev B **90**, 174412 (2014).
20. Zekko, Y. et al. Correlation between the valence state of cerium and the magnetic transition in $Ce(Ru_{1-x}Fe_x)_2Al_{10}$ studied by resonant x-ray emission spectroscopy. Phys Rev. B. **89**, 125108 (2014).
21. Amato, A. et al. Thermopower and magneto-thermopower of $CeRu_2Si_2$ single crystals. J. Magn. Magn. Mater. **76&77**, 263-264 (1988).
